# IRGM/Irgm1 increases autophagy to inhibit activation of NLRP3 inflammasome in inflammatory injury induced acute liver failure

**DOI:** 10.1038/s41420-024-02052-w

**Published:** 2024-06-07

**Authors:** Xing Zhang, Yangyang Hu, Wei Wang, Ru Ji, Ziyue Li, Weiyan Yu, Zhinian Wu, Ying Xiao, Tingyu Guo, Zeqiang Qi, Yadong Wang, Caiyan Zhao

**Affiliations:** Department of Infectious Diseases, the Hebei Medical University Third Hospital, Shijiazhuang, 050051 China

**Keywords:** Liver diseases, Autophagy

## Abstract

Immune-related GTPase M (IRGM) induces autophagy and suppresses inflammation, but its putative role and signaling mechanism remain undefined in the pathogenesis of liver failure. This study aimed to address how IRGM attenuates inflammatory injury by regulating autophagy in liver failure. In this study, a total of 10 patients with hepatitis B virus-related acute-on-chronic liver failure (HBV-ACLF) and 10 healthy controls were prospectively enrolled. Intrahepatic expression of IRGM/Irgm1, NLRP3 inflammasome (NLRP3, ASC, and caspase-1), autophagy-related proteins (LC3II, P62), and inflammatory cytokines (IL-1β, TNF-α) were measured. Autophagy was activated by rapamycin (4 mg/kg) in an acute liver failure (ALF) mouse model, which was used to further study the expression of Irgm1, NLRP3 inflammasome, autophagy-related proteins, and inflammatory cytokines using both qRT-PCR and Western blot analyses. Irgm1 expression was knocked down using Irgm1 short hairpin RNA (shRNA) in lipopolysaccharide (LPS)-induced AML12 cells to investigate the effects of Irgm1 deletion on autophagy and inflammation. We found that the expression of IRGM and autophagy-related proteins was significantly downregulated while the NLRP3 inflammasome was significantly upregulated in the livers of HBV-ACLF patients and the ALF mouse model (all *P* < 0.05). Rapamycin-induced autophagy ameliorated intrahepatic NLRP3 inflammasome activation and decreased inflammation and necrosis in the ALF mice. Irgm1 knockdown decreased autophagy and significantly upregulated NLRP3 inflammasome activation in AML12 cells (all *P* < 0.05). Rapamycin-induced autophagy also protected against hepatocyte injury following LPS stimulation in vitro by inhibiting NLRP3 inflammasome activation. Thus, IRGM/Irgm1 alleviates inflammation-mediated hepatocyte injury by regulating autophagy. This study provides new insight into potential molecular targets to treat liver failure.

## Highlights

- IRGM/Irgm1 alleviates NLRP3-mediated hepatocyte injury by regulating autophagy.
- IRGM/Irgm1 knockdown decreases autophagy and exacerbates activation of the NLRP3 inflammasome.
- Rapamycin protects damaged hepatocytes by restoring the effect of IRGM/Irgm1 downregulation.

## Introduction

Liver failure is a multisystem disorder characterized by jaundice, coagulation disorders, hepatorenal syndrome, hepatic encephalopathy, and ascites with a 30%–70% mortality rate [1–3]. Immune-related inflammatory damage has been proposed as a core pathogenic “driver” of liver failure [4], but its detailed molecular mechanism in the occurrence and development of liver failure requires further investigation. Inflammasome refers to a cytosolic macromolecule consisting of nucleotide-binding oligomerization domain-like receptor 3 (NLRP3), an apoptosis-associated speck-like protein containing C-terminal caspase recruitment domain (ASC), and caspase-1 [5], which acts as an innate immune system sensor that responds to infection and tissue damage. The NLRP3 inflammasome involved in the pathogenesis of viral hepatitis [6–8], non-alcoholic steatohepatitis [8, 9], and alcoholic liver disease [10], can regulate the maturation of interleukin (IL)-1β and IL-18 precursors, triggering liver inflammation. We previously demonstrated that an increase in the NLRP3 inflammasome and its related cytokines (IL-1β, IL-18) in the damaged liver tissue correlates with the severity of hepatic injury caused by hepatitis B virus-related acute-on-chronic liver failure (HBV-ACLF) [6]. Therefore, reducing the inflammatory response by deactivating the NLRP3 inflammasome in liver failure may alleviate tissue damage and improve prognosis. However, in the pathogenesis of liver failure, the regulatory mechanisms of NLRP3 activation need further in-depth study. The research findings will provide important theoretical basis and experimental evidence for exploring the treatment of liver failure by regulating NLRP3 activation.

Autophagy is a cellular process that maintains intracellular homeostasis in response to inflammation and cellular injury [11]. Autophagy has been shown to target the degradation of the NLRP3 inflammasome, subsequently blocking pro-caspase-1 cleavage and activation of IL-1β and IL-18, which attenuates inflammation during liver injury [12]. In addition, autophagy has been shown to be significantly downregulated in the liver tissue of the D-galactosamine/lipopolysaccharide (GaIN/LPS)-induced acute liver failure (ALF) mouse model. Interestingly, it is reported in our previous study that the protective effect of PPARα relies on autophagy-induced inflammatory suppression in ALF [13]. Therefore, targeting autophagy could potentially be an effective treatment for liver failure. In addition, NLRP3 may play an important role in the pathogenesis of autophagy-regulated liver failure.

Immunity-related GTPase M (IRGM) and its mouse orthologue Irgm1 have been proposed as key negative regulators of inflammation and autoimmunity [14, 15]. Although human IRGM and mouse Irgm1 are biochemically different, they are functionally similar in regulating innate immunity and autophagy [16, 17]. IRGM/Irgm1 is associated with several inflammatory and autoimmune diseases, including infectious diseases [16], Crohn’s disease [18], and non-alcoholic fatty liver disease [19]. The regulatory function of IRGM/Irgm1 in autophagy was first described in immune responses against intracellular microorganisms [20]. Further studies demonstrated that IRGM initiated a phosphorylation cascade to activate ULK1 and Beclin-1 to upregulate autophagy [21], as well as interact with NLRP3 and ASC to block assembly and activation of the inflammasome, alleviating inflammatory cell death [22]. However, it remains undefined whether the knockdown of IRGM/Irgm1 can interfere with autophagy and its downstream signaling pathways in liver failure.

This study aimed to test the hypothesis that IRGM/Irgm1 regulates autophagy to inhibit NLRP3 inflammasome activation and pro-inflammatory cytokine release, subsequently preventing or alleviating the progression of liver failure. Therefore, IRGM/Irgm1 was proved to alleviates inflammation-mediated hepatocyte injury by regulating autophagy. This finding provides insight about a potentially new target in the treatment of liver failure.

## Results

### Clinical baseline characteristics of included subjects

A total of 20 subjects, including 10 HBV-ACLF patients and 10 healthy controls (HCs), were recruited in the current study. The HBV-ACLF patients had increased levels of ALT, AST, total bilirubin (TBiL), direct bilirubin (DBiL), international normalized ratio (INR), serum creatinine (SCr), and a higher score of model for end-stage liver disease (MELD), but had decreased levels of serum albumin (ALB) and prothrombin time activity (PTA) compared with the HCs (*P* < 0.05) (Table 1).

**Table 1:** Clinical characteristics of the patients in the different groups.

Characteristic	Healthy controls *n* = 10	HBV-ACLF patients *n* = 10	*t/t’*	*P*-value
Age	35.70 ± 11.01	49.50 ± 15.8	-	>0.05
Male/Female	6/4	7/3	-	>0.05
PLT (×10^9^/L)	213.9 ± 33.11	97.30 ± 33.93	7.38	<0.05
ALB (g/L)	46.90 ± 3.30	28.37 ± 2.33	13.8	<0.05
ALT(U/L)	20.53 ± 7.57	117.13 ± 102.03	−3.58	<0.05
AST(U/L)	22.43 ± 7.79	134.93 ± 130.20	−3.30	<0.05
TBil (μmol/L)	7.66 ± 1.93	255.35 ± 123.51	−12.4	<0.05
DBil (μmol/L)	4.11 ± 1.52	179.17 ± 95.67	−8.13	<0.05
PTA	98.60 ± 5.78	22.50 ± 5.26	29.2	<0.05
INR	0.97 ± 0.10	3.28 ± 0.73	−9.35	<0.05
MELD score	4.50 ± 1.63	31.80 ± 7.59	−10.55	<0.05
Scr (μmol/L)	59.37 ± 8.13	93.02 ± 44.73	−2.22	<0.05

*HBV* hepatitis B virus, *ACLF* acute-on-chronic liver failure, *PLT* platelet, *ALB* albumin, *ALT* alanine aminotransferase, *AST* aspartate aminotransferase, *TBil* total bilirubin, *DBil* direct bilirubin, *PTA* prothrombin time activity, *INR* international normalized ratio, *MELD* model for end-stage liver disease, *SCr* serum creatinine.

### Decreased expression of IRGM and autophagy in HBV-ACLF patients

**Fig. 1:**
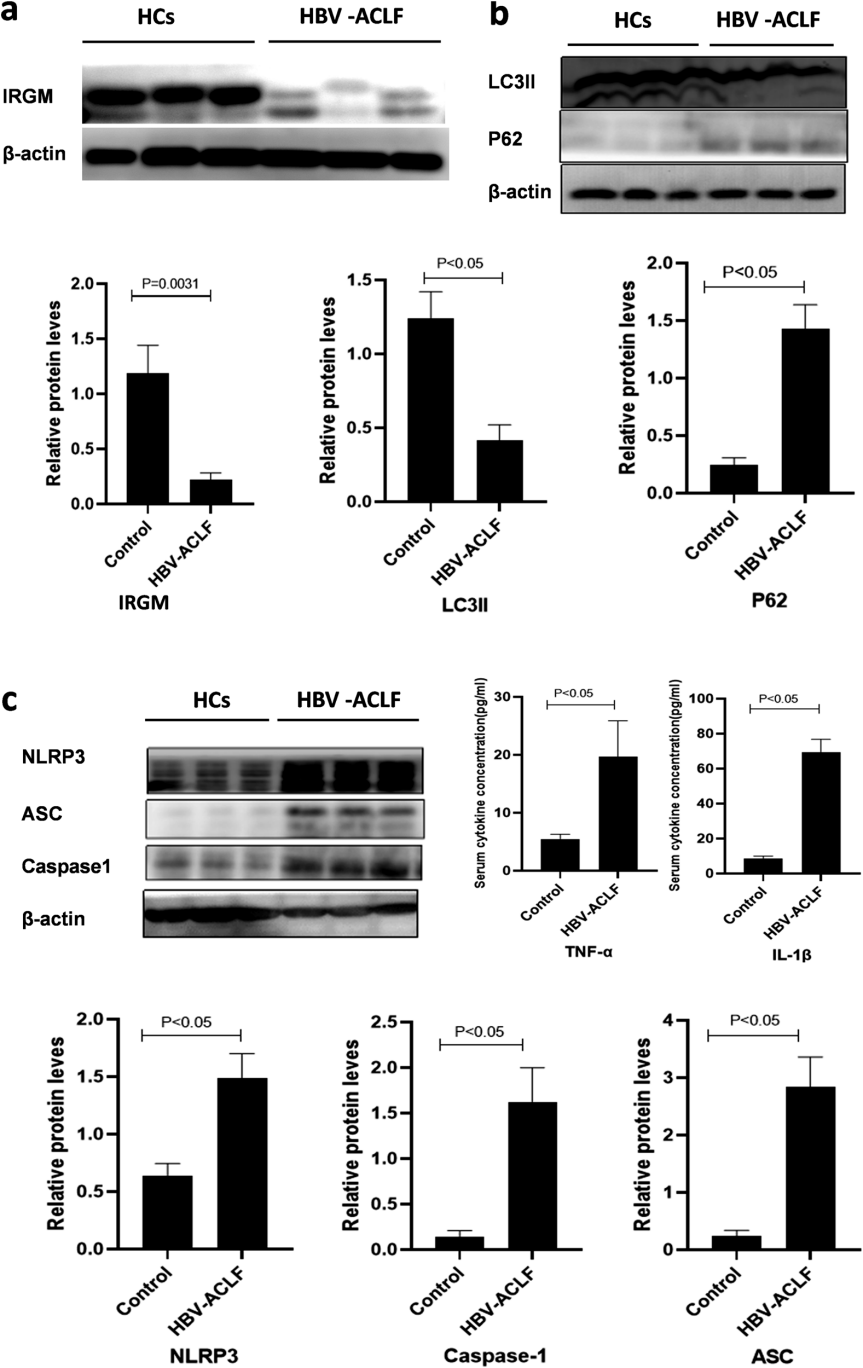
The intrahepatic expression of IRGM, autophagy-related proteins (LC3II, P62), NLRP3, ASC, and caspase-1 in HBV-ACLF patients and HCs. **a** IRGM expression was decreased in HBV-ACLF patients compared to HCs (*P* < 0.05). **b** Expression of LC3II and degradation of P62 were decreased in HBV-ACLF patients compared to HCs (both *P* < 0.05). **c** Intrahepatic expression of NLRP3 inflammasome, ASC, and caspase-1, as well as serum levels of IL-1β and TNF-α were increased in HBV-ACLF patients compared to HCs (all *P* < 0.05). It should be noted that the Western Blot banding randomly selected from three cases of HBV-ACLF patients and HCs in each group were presented.

IRGM protein expression was significantly decreased in the HBV-ACLF patients compared to the HCs (*P* < 0.05, Fig. 1a). There was no significant correlation between IRGM expression and ALT levels, but there were negative correlations between IRGM expression and AST (*r* = −0.52), TBiL (*r* = −0.88), and DBiL (*r* = −0.83) levels (all *P* < 0.05).

Furthermore, the expression of LC3II and degradation of P62 were significantly decreased in the HBV-ACLF patients compared to the HCs (both *P* < 0.05) (Fig. 1b). Pearson correlation analysis further identified that IRGM was positively correlated with LC3II but negatively correlated with P62 (*r* = 0.81, −0.89 respectively, both *P* < 0.05).

### Increased expression of NLRP3 inflammasome and pro-inflammatory cytokines in HBV-ACLF patients

Expression of NLRP3, ASC, caspase-1, and pro-inflammatory cytokines (IL-1β, TNF-α) were all significantly higher in HBV-ACLF patients compared to the HCs (all *P* < 0.05, Fig. 1c). Furthermore, IRGM expression was negatively correlated with the intrahepatic levels of NLRP3, ASC, caspase-1, IL-1β, and TNF-α (*r* = −0.77, −0.82, −0.76, −0.57, −0.52 respectively, all *P* < 0.05), suggesting that increased NLRP3 inflammasome expression was associated with decreased IRGM expression in HBV-ACLF.

### Suppression of intrahepatic expression of Irgm1 in the D-GalN/LPS-induced ALF mouse model

**Fig. 2:**
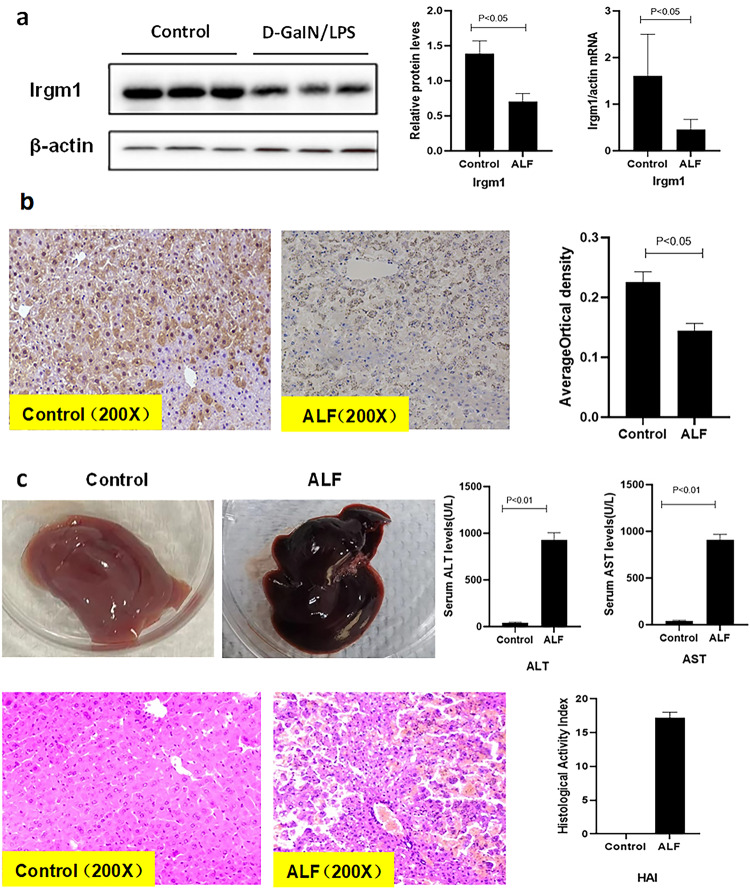
Intrahepatic Irgm1 expression, serum ALT/AST levels, and hepatic histological activity index (HAI) score in ALF mice. **a** Intrahepatic Irgm1 expression significantly decreased in ALF mice compared to controls (both *P* < 0.05). **b** Immunohistochemical staining (200×) showed that the Irgm1 protein was mainly expressed in hepatocytes and that its expression significantly decreased in ALF mice. **c** Representative appearance and H&E staining (200×) showing severe intrahepatic inflammation and necrosis, accompanied by increased hepatic HAI scores and serum ALT and AST levels (all *P* < 0.05).

Massive hepatic injury identified by pathohistological features in the ALF mouse model was apparent 6 h after D-GalN/LPS injection, which met the diagnostic criteria for liver failure. Overall, the 6 h mortality rate was nearly to 80% of ALF mice due to liver failure, and close to 100% of 8 h. Next, we found a decrease in both protein expression and mRNA levels of Irgm1, which were in accordance with the extent of liver injury. Immunohistochemical staining further exhibited the same expression trend of Irgm1 in the livers of the ALF mice. Taken together, these data indicate that Irgm1 was inhibited with the gradual progression of ALF induced by D-GalN/LPS (Fig. 2).

### Increased expression of intrahepatic NLRP3 inflammasome and pro-inflammatory cytokines in the D-GalN/LPS-induced ALF mouse model

**Fig. 3:**
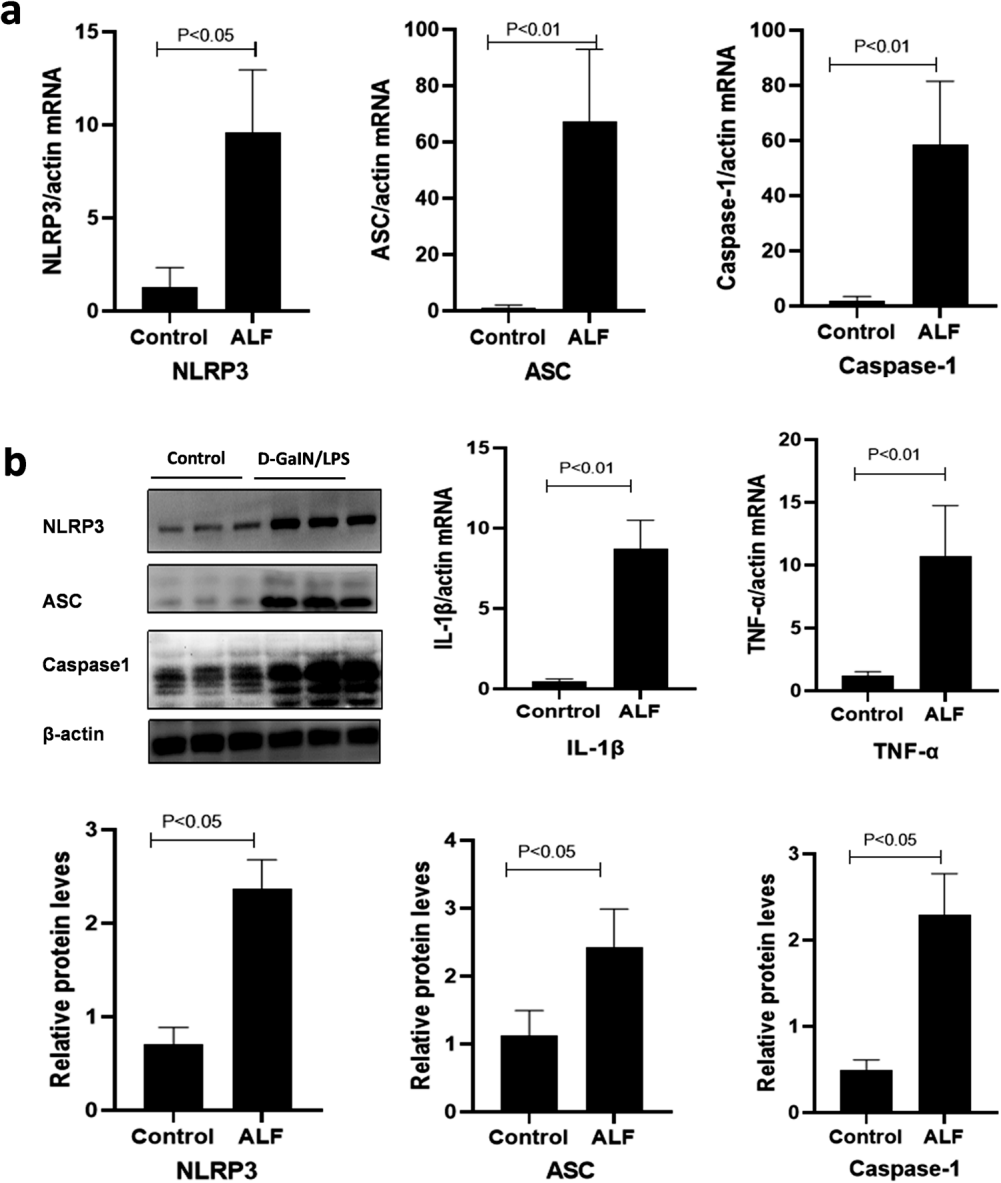
Intrahepatic expression of the NLRP3 inflammasome and pro-inflammatory cytokines in ALF mice. **a** Intrahepatic NLRP3, ASC, and caspase-1 mRNA were increased in ALF mice compared to controls (all *P* < 0.05), which was accompanied by upregulated IL-1β and TNF-α mRNA levels (both *P* < 0.05). **b** Intrahepatic protein expression of NLRP3, ASC, and caspase-1 was increased in ALF mice compared to controls (all *P* < 0.05).

Expression of the NLRP3 inflammasome and pro-inflammatory cytokines was investigated to determine their pathogenic involvement in the progression of ALF. The results showed that the levels of NLRP3, ASC, caspase-1, IL-1β, and TNF-α were significantly higher in the ALF mice compared to the controls (all *P* < 0.05), suggesting that the NLRP3 inflammasome and pro-inflammatory cytokines were elevated in ALF induced by D-GalN/LPS (Fig. 3).

### Enhanced autophagy ameliorates D-GalN/LPS-induced liver injury

**Fig. 4:**
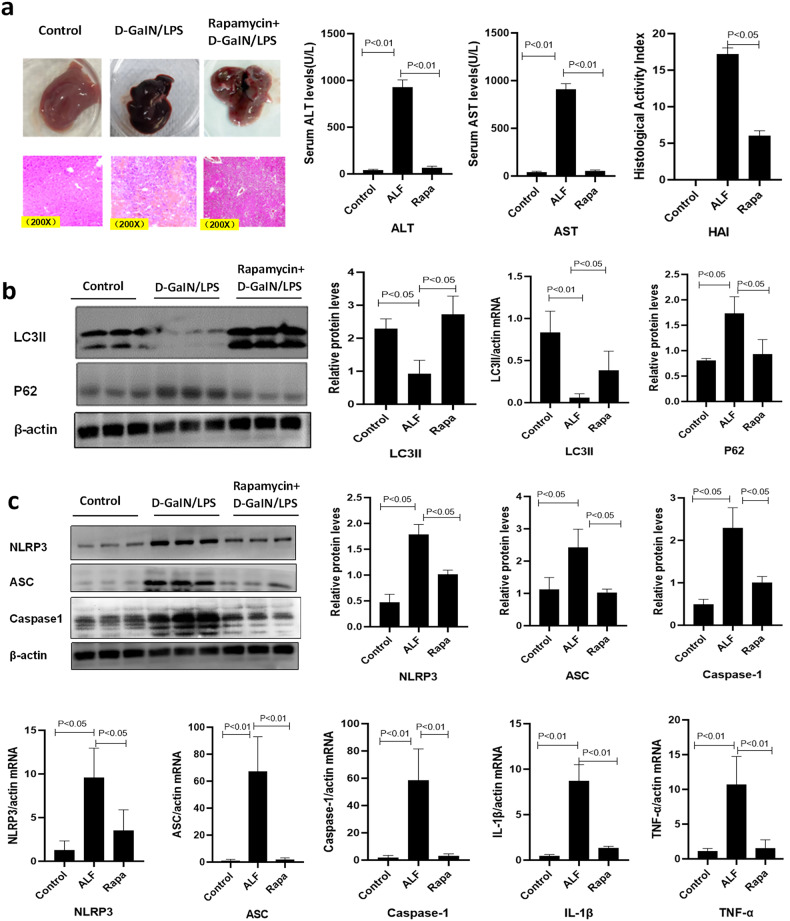
Intrahepatic expression of autophagy-related proteins (LC3II, P62), NLRP3 inflammasome (NLRP3, ASC, caspase-1), and pro-inflammatory cytokines (IL-1β and TNF-α) in ALF mice treated with rapamycin. **a** Reduction of hepatic inflammation and necrosis was evidenced by representative hepatic H&E staining (200×), along with recovered HAI scores and serum ALT and AST levels in ALF mice treated with rapamycin (all *P* < 0.05). **b** Intrahepatic expression of LC3II and degradation of P62 were decreased in ALF mice compared to controls, which were partially recovered by rapamycin (all *P* < 0.05). **c** Intrahepatic expression of NLRP3, ASC, and caspase-1 was increased in ALF mice compared to controls, which were partially recovered by rapamycin. Rapamycin also significantly recovered intrahepatic IL-1β and TNF-α mRNA levels (all *P* < 0.05).

Compared with normal mice, expression of LC3II and degradation of P62 were decreased along with the progression of ALF (both *P* < 0.05). Pretreatment with rapamycin reduced the 6 h mortality from 80% to nearly 25% of ALF model mice. In addition, rapamycin pretreatment preserved the pathohistological morphology and architecture of the liver and decreased inflammatory cell infiltration and hepatocytes necrosis. At the molecular level, rapamycin not only increased LC3II conversion and P62 protein degradation in the D-GalN/LPS-induced ALF mouse model, but also significantly lowered levels of the NLRP3 inflammasome, IL-1β, TNF-α, ALT, and AST compared to mice that were not pretreated with rapamycin (all *P* < 0.05) (Fig. 4).

### Irgm1 knockdown decreases autophagy and exacerbates inflammation in the LPS-induced in vitro hepatocyte injury model

**Fig. 5:**
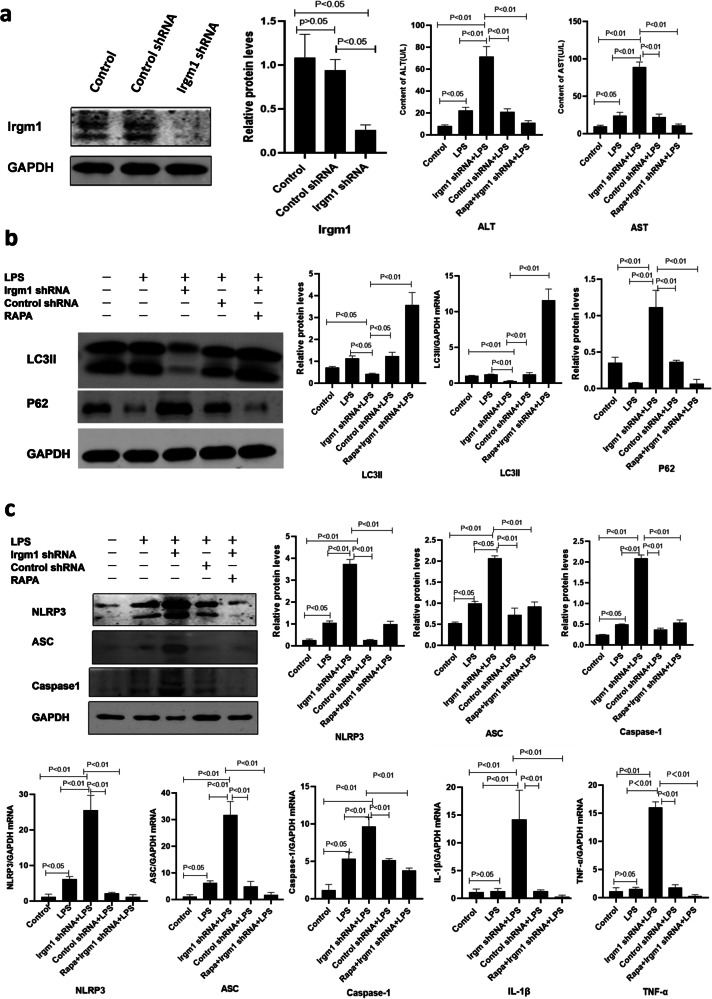
Rapamycin-induced autophagy ameliorates LPS-induced AML12 hepatocyte injury model pretreated with Irgm1 shRNA. **a** Irgm1 knockdown by shRNA decreased the hepatocyte expression of Irgm1 and increased the concentration of ALT and AST in the supernatant, which was significantly decreased by pretreatment with rapamycin (all *P* < 0.05). **b** Increased expression of LC3II and the degradation of P62 in the LPS-induced AML12 hepatocyte injury model were inhibited by Irgm1 shRNA but were significantly reversed by pretreatment with rapamycin (all *P* < 0.05). **c** Expression of NLRP3, ASC, and caspase-1 was significantly increased in the LPS-induced AML12 hepatocyte injury model after Irgm1 knockdown but were significantly decreased by pretreatment with rapamycin (all *P* < 0.05). Compared to controls and the LPS-induced AML12 hepatocyte injury model, the levels of intrahepatic NLRP3, ASC, caspase-1, IL-1β, and TNF-α mRNA were upregulated after Irgm1 knockdown but were significantly recovered by pretreatment with rapamycin (all *P* < 0.05). All cell experiments were averaged from triplicate wells and repeated five times.

After 12 h of intervention with LPS, the ALT and AST levels in the supernatant from the LPS-induced AML12 hepatocyte injury cell model were significantly increased compared to the control cells. LPS treatment also increased the levels of the NLRP3 inflammasome, IL-1β, and TNF-α in the cultured hepatocytes. Irgm1 knockdown by shRNA decreased LC3II conversion and P62 protein degradation compared to the control and LPS-treated cells (all *P* < 0.05). Furthermore, the levels of ALT and AST were significantly elevated in the supernatant of the cells treated with Irgm1 shRNA and LPS compared to the other groups (all *P* < 0.05). In addition, the levels of NLRP3, ASC, caspase-1, IL-1β, and TNF-α were significantly higher in the Irgm1 shRNA+LPS group than those in the control and LPS-treated groups (all *P* < 0.05) (Fig. 5).

### Enhanced autophagy ameliorates hepatocyte injury induced by LPS

To confirm whether the protective effect of Irgm1 was involved in attenuating inflammation by regulating autophagy, we pretreated the Irgm1 shRNA+LPS cells with rapamycin. Pretreatment with rapamycin enhanced LC3II conversion and P62 protein degradation in the LPS-induced hepatocytes but normalized the levels of ALT and AST in the supernatant compared to the cells that were not pretreated with rapamycin (all *P* < 0.05). Moreover, levels of NLRP3, ASC, caspase-1, IL-1β, and TNF-α were significantly decreased in the rapamycin treatment group compared to the group that was not treated with rapamycin (all *P* < 0.05) (Fig. 5).

## Discussion

Liver failure induced by various factors results in severe loss of liver function [1, 2], which is often accompanied by life-threatening complications such as hemorrhage and hepatic encephalopathy. This study showed elevations in ALT and AST serum levels in both HBV-ACLF patients and ALF mice induced by D-GalN/LPS, along with extensive hepatocyte edema and necrosis in large patches, infiltration of inflammatory cells, diffuse biliary stasis, and collapsed fibrous meshwork scaffolds, suggesting inflammatory necrosis of hepatocytes is important in the pathogenesis of liver failure.

Previous studies found that activation of the NLRP3 inflammasome may participate in the pathogenesis of ALF. Li et al. [23] reported that expression of NLRP3, ASC, IL-1β, IL-18, and sCD40L was increased in patients with HBV-ACLF. Moreover, pro-caspase-1 and pro-IL-1β, components of the NLRP3 inflammasome machinery, were also upregulated. Our study observed similar changes in the NLRP3 inflammasome in the livers of HBV-ACLF patients and in the D-GaIN/LPS-induced ALF mouse model. Therefore, inflammation mediated by excessive activation of the NLRP3 inflammasome can be a putative factor that induces hepatocyte injury in ALF. Wang et al. [24] used Isoliquiritigenin to inhibit the NLRP3 inflammasome, consequently preventing the development of D-GalN/LPS-induced ALF. Verapamil has also been shown to alleviate ALF by inhibiting the thioredoxin interacting protein (TXNIP)/NLRP3 pathway [25]. Taken together, targeting NLRP3 may be a potential strategy in the treatment of ALF.

Inflammatory responses are associated with pathogenetic mechanisms of liver failure and can be regulated by many factors, among which autophagy functions to digest misfolded proteins and damaged organelles to maintain internal environment homeostasis. Even though a few studies have suggested that autophagy may be involved in the pathogenesis of liver failure, the exact mechanism remains undefined. A growing body of evidence found that autophagic enhancement alleviated immune inflammatory damage in ALF. Lin et al. [26] demonstrated that Eva1a-mediated autophagy attenuated inflammatory responses and apoptosis to ameliorate liver injury in ALF mice. Jiao et al. [13] reported that PPARα-mediated autophagic induction could lessen inflammation conducive to liver injury. In addition, it has been reported that there are changes in autophagic flux during the progression of liver failure [27]. The reasons for why changes in autophagy vary in liver failure, or even why there are opposing findings in the literature, may be due to the different pathologic phases or injury severity of liver failure. The present study showed that autophagy was suppressed manifested by the decreased LC3II but increased P62 expression in different degrees due to the downregulated conversion from LC3I to LC3II and the suppressed degradation of P62 in the HBV-ACLF patients and the ALF mice, which was congruent with NLRP3 inflammasome activation and the increase in IL-1β and TNF-α synthesis and release. This finding suggests that autophagy is protective and determines inhibition of NLRP3 inflammasome activation and subsequent pro-inflammatory cytokine synthesis and release, which is based on the pathophysiological activation of autophagy that was shown to prevent mice from developing ALF after D-GalN/LPS induction. This correlation was further confirmed in cells pretreated with rapamycin to enhance autophagy, deactivate the NLRP3 inflammasome, and downregulate the release of pro-inflammatory cytokines. Interestingly, in our in vitro studies, the degree of autophagy was increased in the LPS-induced hepatocyte injury model, and there was an increase in NLRP3 inflammasome activation and IL-1β and TNF-α synthesis and release. These findings contrast the in vivo results in the ALF mice. This contradiction may be due to variant hepatocytes injury degree in the different stages of liver failure between the injured liver cell model and the ALF mouse model, which was also identified by previous research of Shen et al. [28]. These results further suggest that the autophagic flow may exist and is important in the occurrence and development of liver failure. Altogether, autophagy may protect against the development and progression of ALF through attenuating excessive NLRP3 inflammasome activation, although the autophagic flow may exist and the protective effect may be inhibited and even lost in advanced liver failure. Thus, targeting autophagy may be a potential strategy for treating early-stage liver failure. The specific signaling pathway and molecular mechanism by which autophagy regulates NLRP3 inflammasome activation as well as the temporal variations in autophagy and autophagic flux in the pathogenesis of liver failure need to be further explored.

Previous studies identified an association of IRGM/Irgm1 with autophagy. Jean et al. [29] and Nath et al. [30] claimed that IRGM/Irgm1 interacted with nucleic acid sensor proteins, cGAS and RIG-I, to regulate P62-dependent autophagic degradation to suppress interferon signaling. Lin et al. [31] found that IRGM knockdown inhibited autophagic flux along with increased lipid droplet content in HepG2 and PLC/PRF/5 cells, which were corrected by the administration of rapamycin. The present study further showed that IRGM/Irgm1 expression was not only inhibited in patients with HBV-ACLF and in the ALF mouse model, but it also correlated with the severity of hepatic injury. Thus, it has been hypothesized that IRGM/Irgm1, as an important upstream regulatory molecule, is involved in regulating autophagy in the pathogenesis of liver failure. We further utilized the LPS-induced AML12 hepatocyte injury cell model to confirm that IRGM/Irgm1 expression was downregulated and autophagy was changed accordingly in in vitro models of liver injury. To confirm the molecular mechanism of IRGM/Irgm1 regulation of liver failure, we knocked down Irgm1 expression in vitro to measure gene regulation following LPS-induced hepatocyte injury. Irgm1 knockdown by shRNA decreased autophagic activities but upregulated inflammation in the LPS-induced AML12 cell injury model. However, rapamycin was able to partly restore the effect of Irgm1 knockdown, enhance autophagy activation, and protect hepatocytes against LPS-induced injury. Although the Control+ rapamycin alone treatment group was not set up in this study to verify the intensity changes of autophagy and the following effects after rapamycin intervention in normal hepatocytes, it does not affect the finding that rapamycin improved the inflammation degree and hepatocytes injury by restoring the effect of Irgm1 knockdown induced autophagy inhibition. Collectively, these findings suggest that IRGM/Irgm1 can regulate autophagy to inhibit the activation of the NLRP3 inflammasome, as well as the production and release of pro-inflammatory cytokines, which protect against the pathogenesis of liver failure.

In summary, this study confirmed that IRGM/Irgm1 functions as a part of the machinery that protects against liver failure by upregulating autophagy and reducing liver inflammation. This study provides new experimental evidence for exploring autophagy and inflammatory regulation in liver failure. As an important regulator of autophagy and inhibitor of inflammation in liver failure, IRGM/Irgm1 could be considered a potential molecular target for treating liver failure. Although, there are some defects in this study, such as the enrollment sample of clinical study is limited due to difficulty to obtain samples from patients with liver failure; the relationship of NLRP3 inflammasome activation and decreased IRGM expression is only based on correlation analysis, but no research evidence was provided for that direct association; the molecular mechanism about IRGM1 induces autophagy and inhibits NLRP3 inflammasome activation is too simplistic, and even future validation experiments in Irgm1, NLRP3 or LC3II KO mice models are needed to confirm these findings. But there is no doubt developing molecular therapeutics that target IRGM/Irgm1 may become an important strategy in the treatment and prevention of liver failure. Next, we will go deep into further research.

## Material and methods

### Collection and preparation of clinical specimens

Blood samples and liver tissue specimens were separately collected from 10 HBV-ACLF patients receiving liver transplantations. In addition, liver tissue specimens of 10 HCs were collected from discarded liver tissue during the clipping and repairing process the donor liver as liver transplantation, and the blood samples from 10 healthy blood donation volunteers. HBV-ACLF was diagnosed based on the consensus recommendation for ACLF criteria (2014) by the Asian Pacific Association for the Study of the Liver (APASL). The study protocol was approved by the Human Research Ethics Committee of our hospital. All procedures were conducted in accordance with the Declaration of Helsinki. All patients or their close relatives provided written informed consent before participating in the study.

### ALF mouse model

Male C57BL/6 mice aged 8–12 weeks were housed in a 12:12 light-dark cycle with a cage temperature of 22 ± 1 °C and fed water and normal diets ad libitum. In accordance with the random number table, they were then randomly divided into the healthy control group (*n* = 8), ALF model group (*n* = 8), and the RAPA treatment group (*n* = 8). The ALF model group mice received intraperitoneal injection of D-GalN (500 mg/kg; Sigma, USA) and LPS (10 μg/kg; Sigma, USA) to induce ALF. To induce autophagy, rapamycin (4 mg/kg; Sigma, USA) was intraperitoneally injected into the RAPA treatment group mice 1 h before the administration of D-GalN/LPS. The mice were sacrificed 6 h later, and liver specimens and serum samples were collected for future detection. The animal study was approved by the Laboratory Animal Ethical and Welfare Committee of the Hebei Medical University.

### Histopathological and immunohistochemical analysis

The liver specimens were fixed with 4% paraformaldehyde, embedded in paraffin wax, and sectioned into 3 μm-thick slices. Then, the tissue sections were stained with hematoxylin and eosin (H&E) and visualized using light microscopy to identify hepatic inflammation.

Liver tissue sections were deparaffinized, rehydrated, and subjected to antigen retrieval. Afterward, the specimens were incubated with primary polyclonal rabbit-anti-human antibodies (1:200) (Cell Signaling, USA) at 4 °C overnight, and then incubated in a goat-anti-rabbit secondary antibody (Proteintech Group, China) for 40 min at 37 °C. The staining was developed using a DAB substrate and counter-stained with hematoxylin. Lastly, the samples were dehydrated and sealed with coverslips for blinding microscopic analysis by two pathologists major in hepatology.

### Establishment of in vitro hepatocyte injury

AML12 cells, purchased from Guangzhou Saiku Biotechnology Co., Ltd. were cultured in DMEM/F12 medium with 10% fetal bovine serum, 1% penicillin, and 1% streptomycin in an incubator at 37 °C, 5% CO_2_, and 95% humidity. The cells were harvested at the logarithmic growth stage (the 3rd–10th generations) and then transferred onto cell culture plates. When the cells reached 50%–70% confluence, they were treated with LPS (1 μg/mL) for 12 h to establish the in vitro hepatocyte injury model.

### Knockdown of Irgm1 using short hairpin RNA (shRNA)

Irgm1 and control shRNA vectors (Shanghai Genechem, China) were separately transfected into the AML12 cells with Lipofectamine 2000 (Invitrogen, USA). In brief, once the cells reached 60%–70% confluence, they were transfected with a mixture of 2 μL shRNA (20 mM) and 2 μL Lipo2000. Irgm1 gene silencing was evaluated using Western blot analysis 48 h after transfection to select the most efficient construct for subsequent experiments.

### Western blot analysis

Proteins were extracted from liver tissues (from each clinical specimen and mouse liver tissue specimen) or AML12 cells (each group including 5 Wells, and the experimental detection was repeated for 3 times) in RIPA buffer (Solarbio, China), and separated using SDS-12% polyacrylamide gel electrophoresis. The separated proteins were then transferred to PVDF membranes (Bio-Rad, USA) and blocked with 5% skimmed milk in Tris-buffered saline with Tween 20 for 2 h. Afterward, the membranes were incubated with primary antibodies (1:1000) against IRGM/Irgm1 (40kD/20kD, 71950, NBP1-76377), LC3I/II (14/16kD, 12741), P62 (62kD, 5114), NLRP3 (110kD, 15101), ASC (24kD, sc-514414), caspase-1 (43kD, 22915-1-AP), and β-actin/GAPDH (45kD/36kD, 4970, Cell Signaling, USA, Novus Biologicals, USA, Proteintech, China, Santa Cruz Biotechnology, USA) at 4 °C overnight, then thoroughly washed and incubated with a horseradish peroxidase-conjugated secondary antibody (1:8000) for 1 h at room temperature. The protein bands were detected using an enhanced luminescent developer (Millipore) and imaged using a ChemiDoc MP System (Bio-Rad, USA). The gray values of the bands were quantitatively measured using ImageJ software.

### Quantitative real-time PCR analysis (qRT-PCR)

Total RNA was extracted using Trizol reagent (Invitrogen, USA) from liver tissues and AML12 cells (instructions for grouping and specimen collection were the same as Western blot analysis). A total of 2 ug of RNA was reverse transcribed into cDNA using the SuperScript III First-Strand Synthesis System (Invitrogen, USA). Quantitative PCR was done in the DNA Engine with Chromo 4 Detector (MJ Research, USA). The reactants were mixed at a final volume of 20 μl as follows: 1× SuperMix from Platinum SYBR Green qPCR Kit (Invitrogen, USA); cDNA (2 μl); and 0.5 μM of each primer for Irgm1, LC3II, NLRP3, caspase-1, ASC, IL-1β, and tumor necrosis factor (TNF)-α. PCR was done using the follow conditions: 95 °C for 10 min, followed by 40 cycles of 95 °C for 15 s, and 60 °C for 1 min. The relative mRNA levels were normalized to β-actin/GAPDH, and data were analyzed using the 2^−ΔΔCt^ method. The primers used to amplify the respective mouse gene fragments are listed in Table 2.

**Table 2:** Primers used for real-time PCR.

Target gene	Forward primers	Reverse primers
Irgm1	TGGCAATGGCATGTCATCTT	AGTACTCAGTCCGCGTCTTCGT
LC3II	CAAGCCTTCTTCCTCCTGGTGAATG	CCATTGCTGTCCCGAATGTCTCC
NLRP3	ACGACGAGGCCGGTAGCCTT	TTCTGCAGGCCGTGCTTCCG
ASC	CTTGTCAGGGGATGAACTCAAAA	GCCATACGACTCCAGATAGTAGC
caspase-1	ACAAGGCACGGGACCTATG	TCCCAGTCAGTCCTGGAAATG
IL-1β	TTGACGGACCCCAAAAGAT	GATGATCTGAGTGTGAGGGTCTG
TNF-α	GCCTCTTCTCATTCCTGCTTGT	TTGAGATCCATGCCGTTG
β-actin	CTACCTCATGAAGATCCTGACC	CACAGCTTCTCTTTGATGTCAC

### ELISA analysis

IL-1β and TNF-α levels in the serum were measured using ELISA kits according to the manufacturer’s instructions (ExCell Biology Inc., China).

### Statistical analysis

SPSS 21.0 software was used for statistical analysis. When the data were normally distributed and the variances were homogeneous, an independent sample *t*-test was used to compare two groups, one-way ANOVA analysis of variance was used to compare multiple groups, and Fisher’s LSD method was used to compare differences between two groups. When the data were not normally distributed or uneven variance existed, the *t’* test was used to compare differences between two groups, the Kruskal–Wallis H rank sum test was used to compare differences among multiple groups, and the two-sample Mann–Whitney *U*-test was used to compare differences between two groups. Data were subjected to Pearson correlation analysis. Two-sided *P*-value < 0.05 was considered statistically significant.

### Supplementary information

Supplementary Material

## Data Availability

The datasets generated and analyzed during the current study are available from the corresponding author on reasonable request.
