# Supplementary material for: IRGM/Irgm1 increases autophagy to inhibit activation of NLRP3 inflammasome in inflammatory injury induced acute liver failure
Source: Cell Death Discov. 2024 Jun 7;10:272. doi: 10.1038/s41420-024-02052-w (PMC11161524; doi:10.1038/s41420-024-02052-w)
Supplement: Supplementary file 1 — Supplementary Material [file 41420_2024_2052_MOESM1_ESM.pptx]

## Slide 1
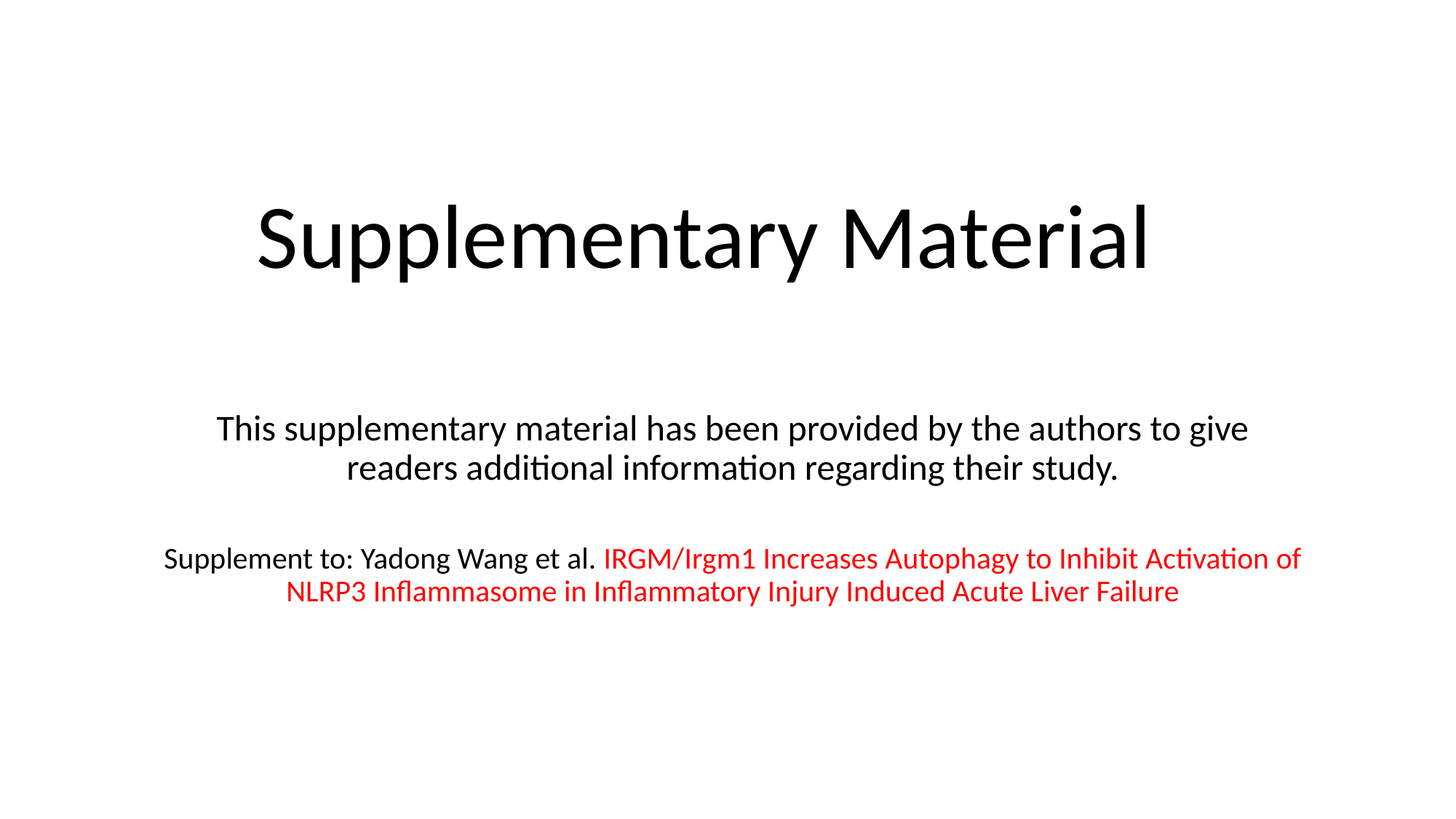

# Supplementary Material
This supplementary material has been provided by the authors to give readers additional information regarding their study.
Supplement to: Yadong Wang et al. IRGM/Irgm1 Increases Autophagy to Inhibit Activation of NLRP3 Inflammasome in Inflammatory Injury Induced Acute Liver Failure

## Slide 2
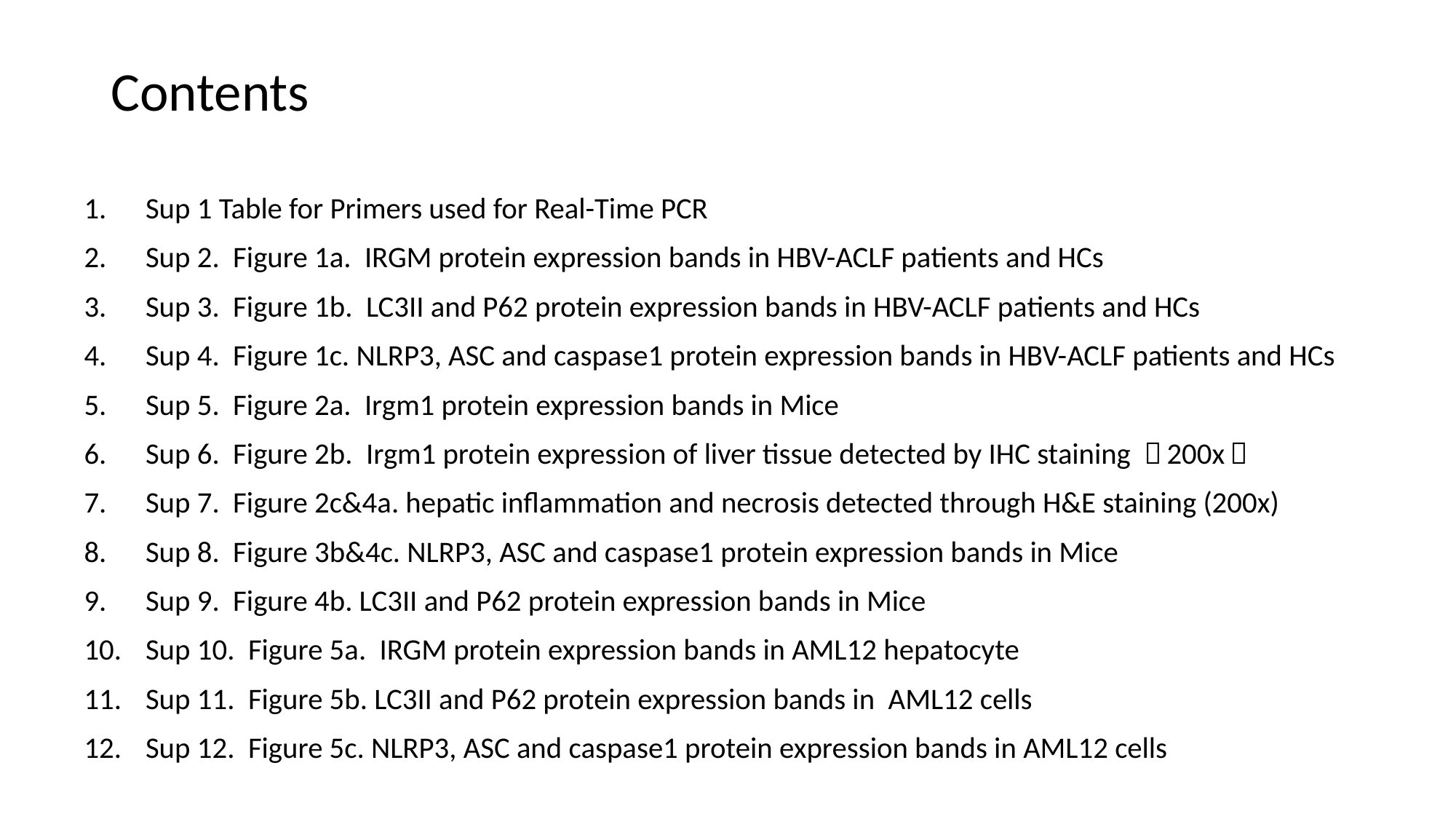

# Contents
Sup 1 Table for Primers used for Real-Time PCR
Sup 2. Figure 1a. IRGM protein expression bands in HBV-ACLF patients and HCs
Sup 3. Figure 1b. LC3II and P62 protein expression bands in HBV-ACLF patients and HCs
Sup 4. Figure 1c. NLRP3, ASC and caspase1 protein expression bands in HBV-ACLF patients and HCs
Sup 5. Figure 2a. Irgm1 protein expression bands in Mice
Sup 6. Figure 2b. Irgm1 protein expression of liver tissue detected by IHC staining （200x）
Sup 7. Figure 2c&4a. hepatic inflammation and necrosis detected through H&E staining (200x)
Sup 8. Figure 3b&4c. NLRP3, ASC and caspase1 protein expression bands in Mice
Sup 9. Figure 4b. LC3II and P62 protein expression bands in Mice
Sup 10. Figure 5a. IRGM protein expression bands in AML12 hepatocyte
Sup 11. Figure 5b. LC3II and P62 protein expression bands in AML12 cells
Sup 12. Figure 5c. NLRP3, ASC and caspase1 protein expression bands in AML12 cells

## Slide 3
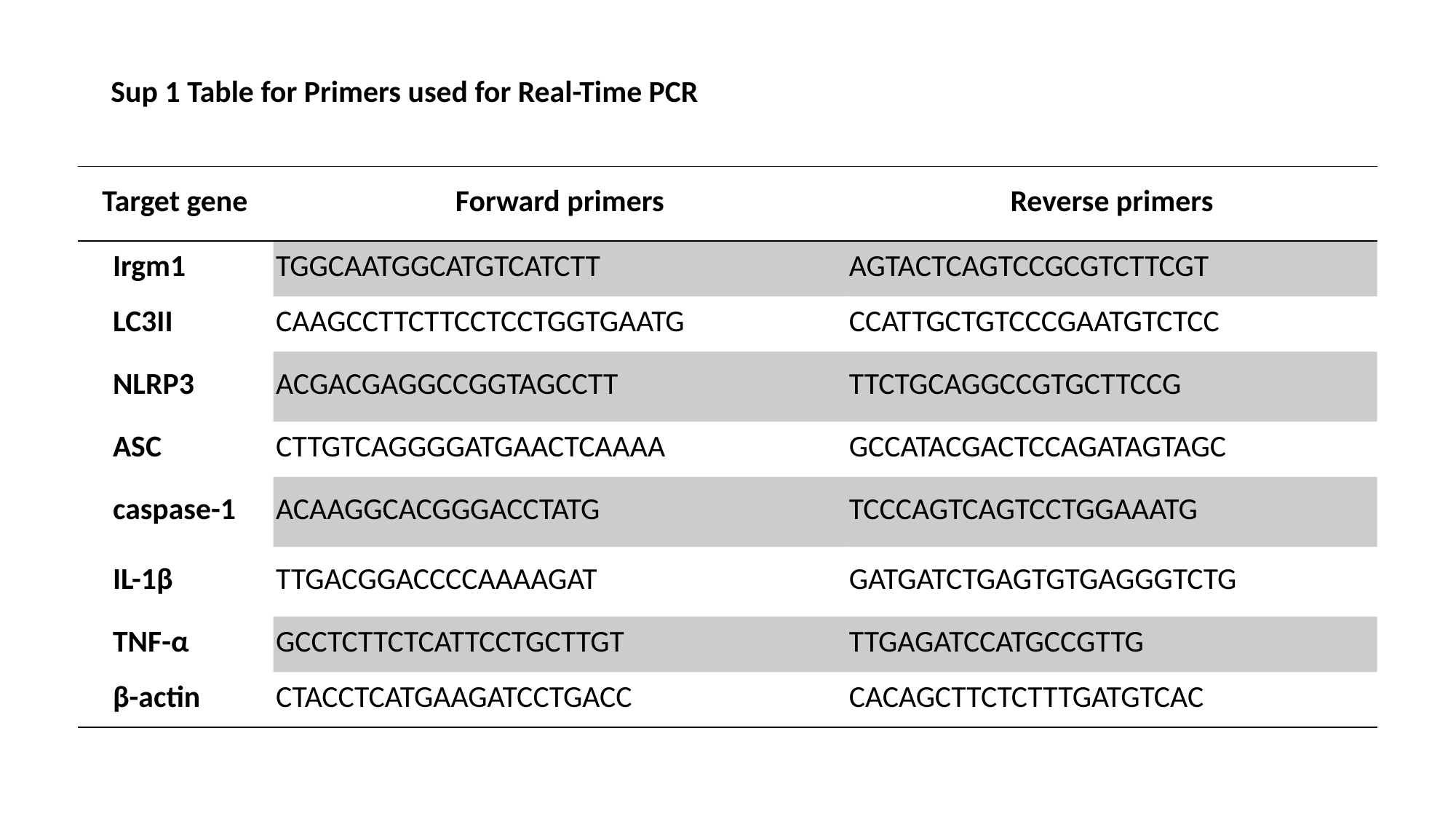

# Sup 1 Table for Primers used for Real-Time PCR
| Target gene | Forward primers | Reverse primers |
| --- | --- | --- |
| Irgm1 | TGGCAATGGCATGTCATCTT | AGTACTCAGTCCGCGTCTTCGT |
| LC3II | CAAGCCTTCTTCCTCCTGGTGAATG | CCATTGCTGTCCCGAATGTCTCC |
| NLRP3 | ACGACGAGGCCGGTAGCCTT | TTCTGCAGGCCGTGCTTCCG |
| ASC | CTTGTCAGGGGATGAACTCAAAA | GCCATACGACTCCAGATAGTAGC |
| caspase-1 | ACAAGGCACGGGACCTATG | TCCCAGTCAGTCCTGGAAATG |
| IL-1β | TTGACGGACCCCAAAAGAT | GATGATCTGAGTGTGAGGGTCTG |
| TNF-α | GCCTCTTCTCATTCCTGCTTGT | TTGAGATCCATGCCGTTG |
| β-actin | CTACCTCATGAAGATCCTGACC | CACAGCTTCTCTTTGATGTCAC |

## Slide 4
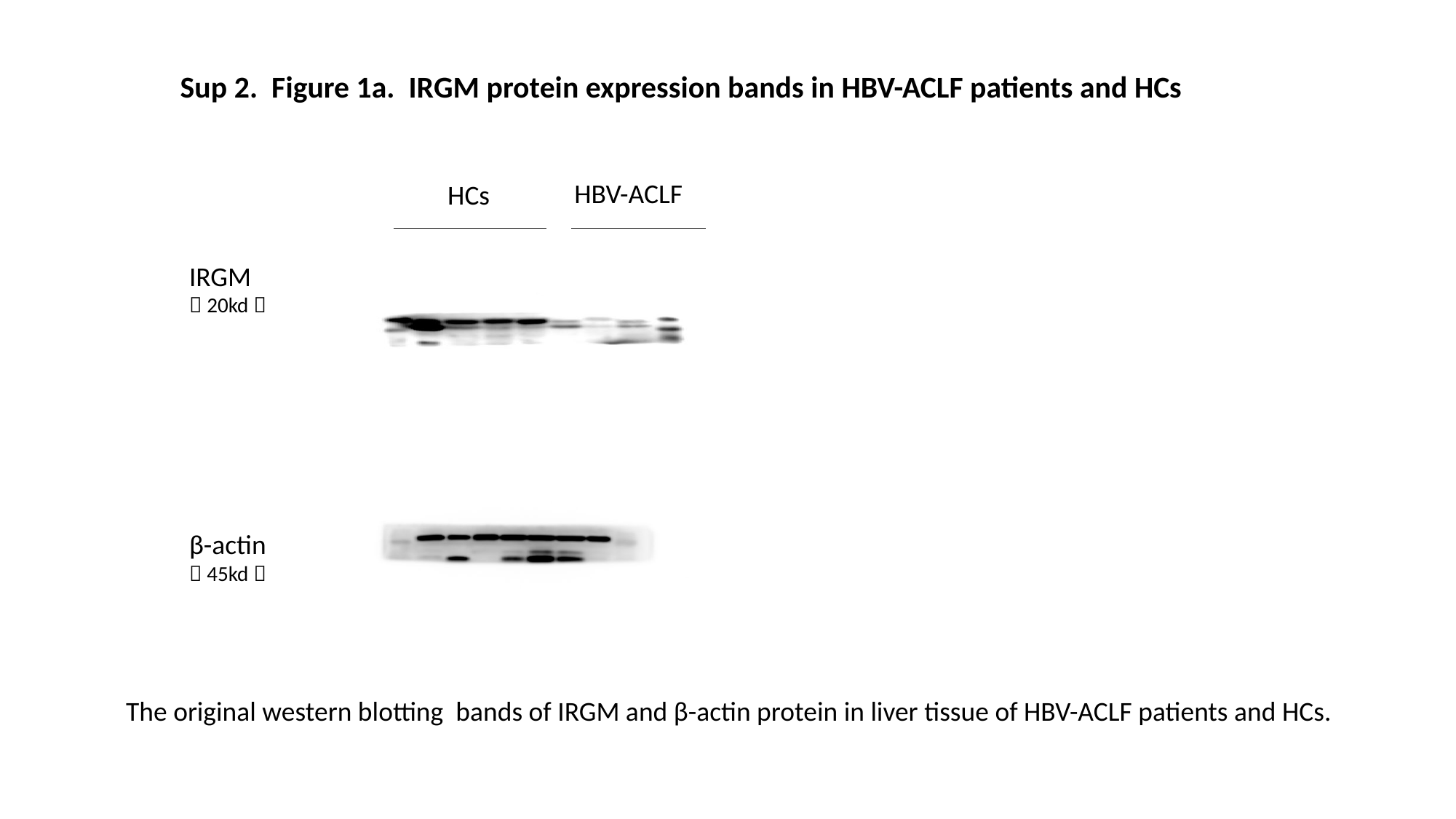

Sup 2. Figure 1a. IRGM protein expression bands in HBV-ACLF patients and HCs
HBV-ACLF
HCs
IRGM
（20kd）
β-actin
（45kd）
The original western blotting bands of IRGM and β-actin protein in liver tissue of HBV-ACLF patients and HCs.

## Slide 5
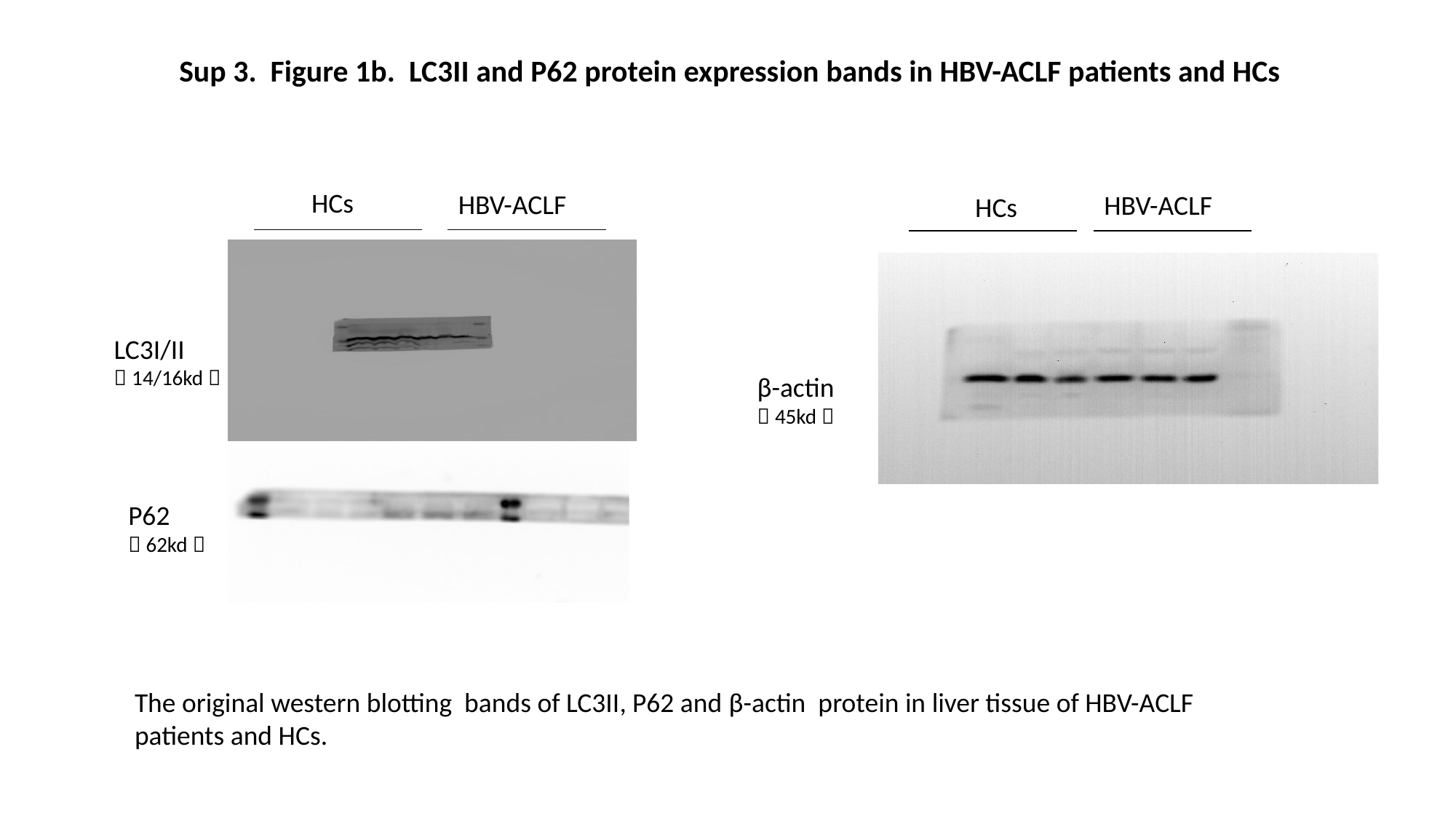

Sup 3. Figure 1b. LC3II and P62 protein expression bands in HBV-ACLF patients and HCs
HCs
HBV-ACLF
HBV-ACLF
HCs
LC3I/II
（14/16kd）
β-actin
（45kd）
P62
（62kd）
The original western blotting bands of LC3II, P62 and β-actin protein in liver tissue of HBV-ACLF patients and HCs.

## Slide 6
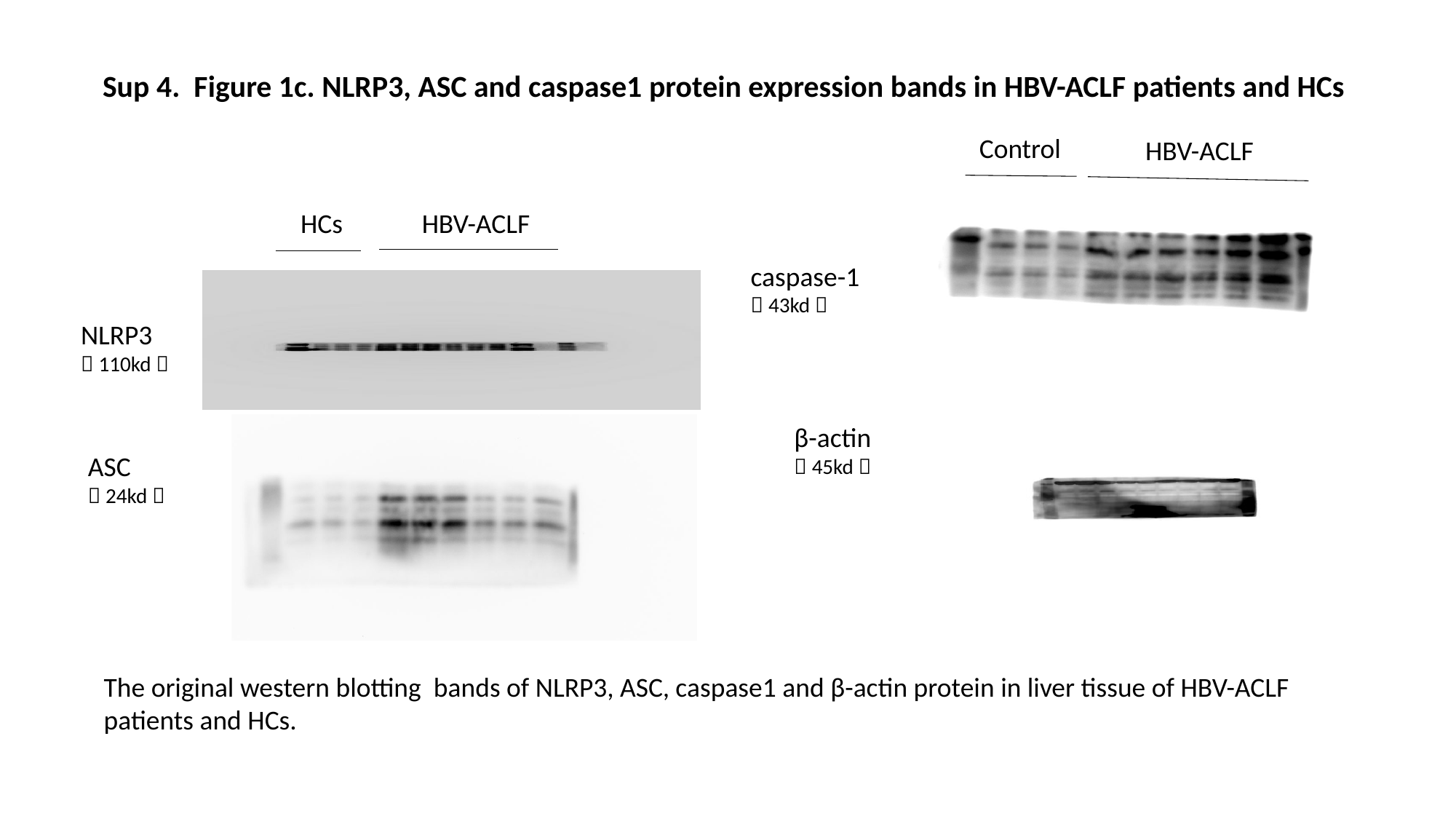

Sup 4. Figure 1c. NLRP3, ASC and caspase1 protein expression bands in HBV-ACLF patients and HCs
Control
HBV-ACLF
HCs
HBV-ACLF
caspase-1
（43kd）
NLRP3
（110kd）
β-actin
（45kd）
ASC
（24kd）
The original western blotting bands of NLRP3, ASC, caspase1 and β-actin protein in liver tissue of HBV-ACLF patients and HCs.

## Slide 7
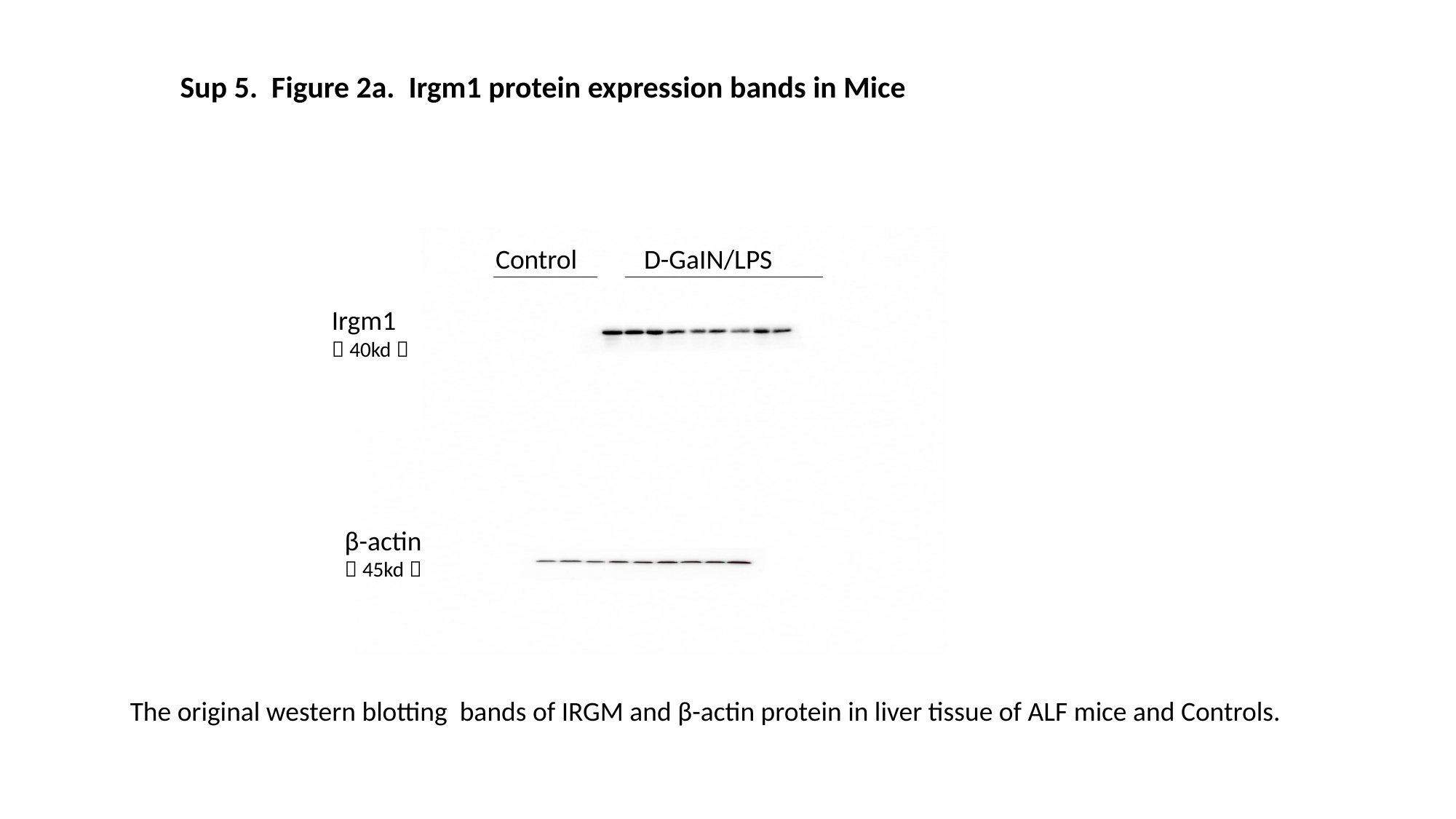

Sup 5. Figure 2a. Irgm1 protein expression bands in Mice
Control
D-GaIN/LPS
Irgm1
（40kd）
β-actin
（45kd）
The original western blotting bands of IRGM and β-actin protein in liver tissue of ALF mice and Controls.

## Slide 8
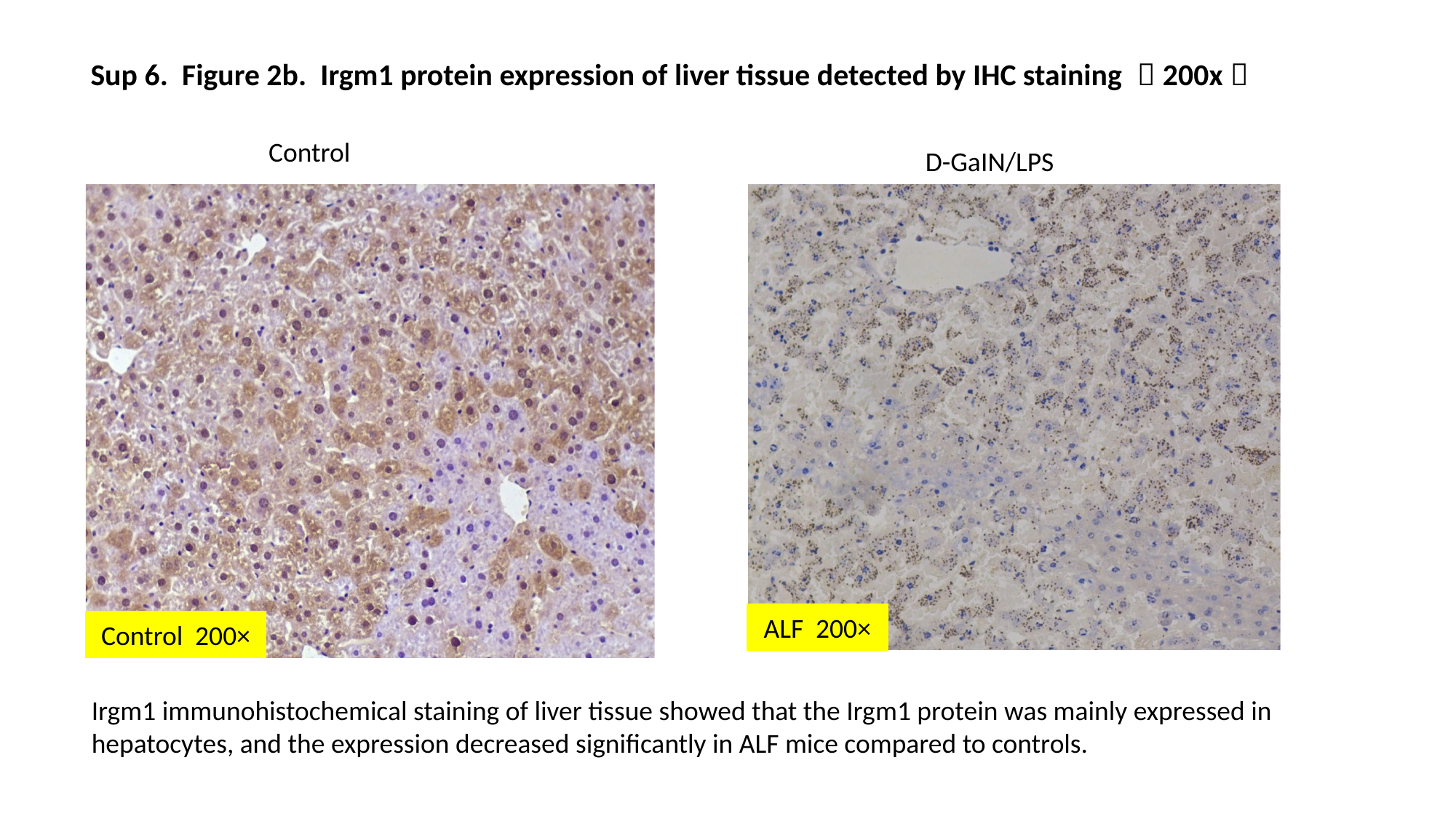

Sup 6. Figure 2b. Irgm1 protein expression of liver tissue detected by IHC staining （200x）
Control
D-GaIN/LPS
ALF 200×
Control 200×
Irgm1 immunohistochemical staining of liver tissue showed that the Irgm1 protein was mainly expressed in hepatocytes, and the expression decreased significantly in ALF mice compared to controls.

## Slide 9
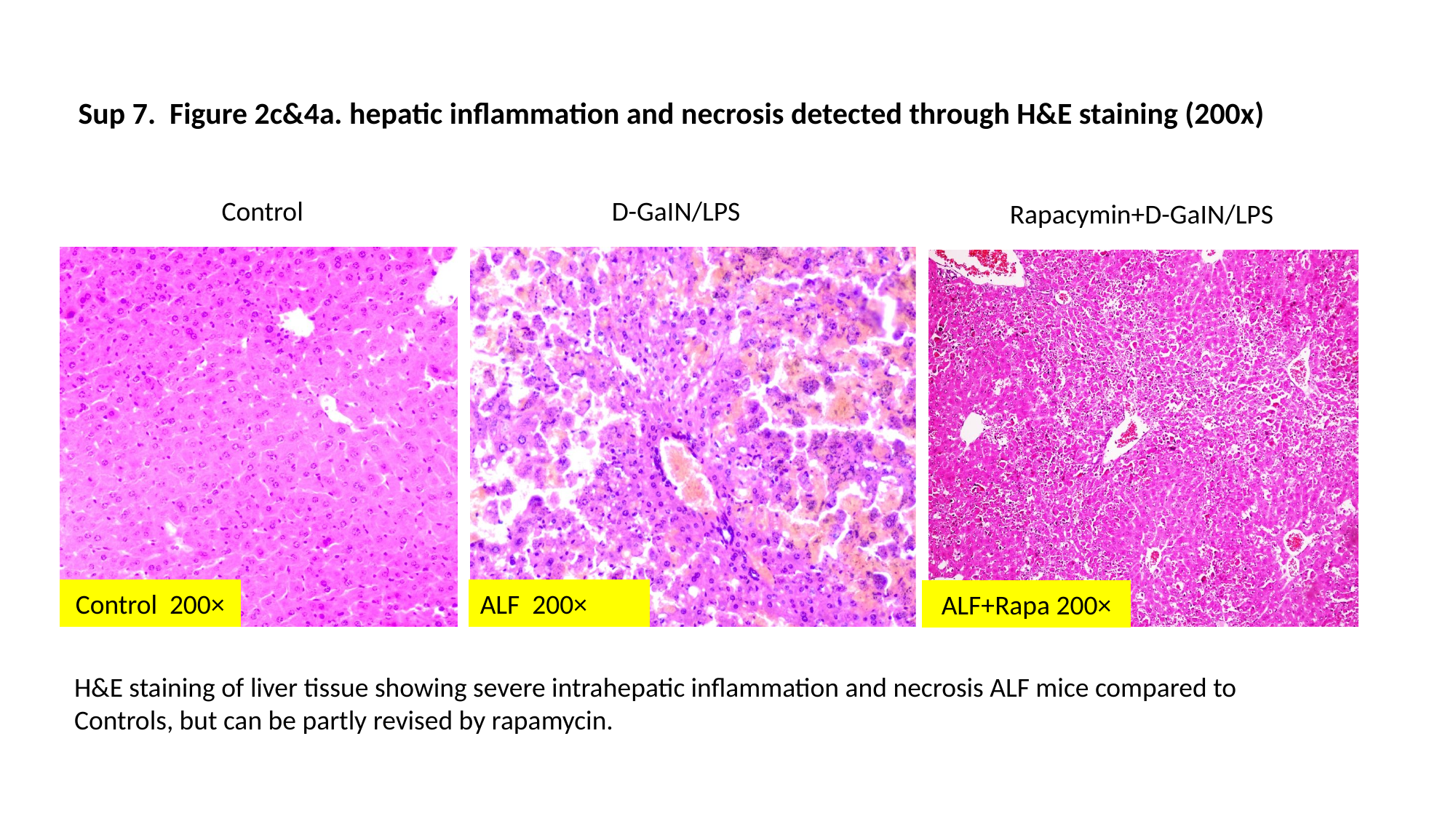

Sup 7. Figure 2c&4a. hepatic inflammation and necrosis detected through H&E staining (200x)
Control
D-GaIN/LPS
Rapacymin+D-GaIN/LPS
Control 200×
ALF 200×
ALF+Rapa 200×
H&E staining of liver tissue showing severe intrahepatic inflammation and necrosis ALF mice compared to Controls, but can be partly revised by rapamycin.

## Slide 10
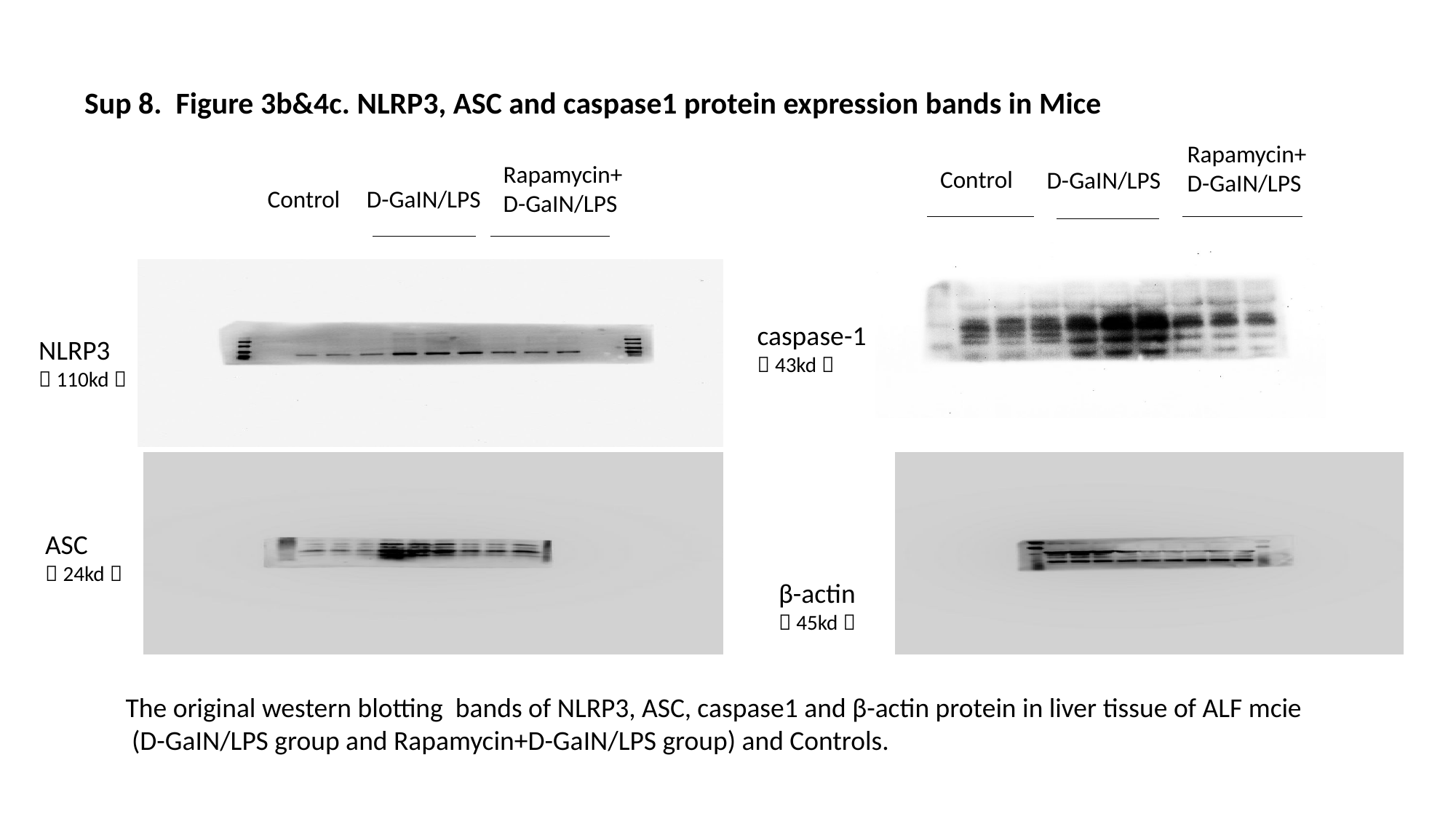

Sup 8. Figure 3b&4c. NLRP3, ASC and caspase1 protein expression bands in Mice
Rapamycin+
D-GaIN/LPS
Rapamycin+
D-GaIN/LPS
Control
D-GaIN/LPS
Control
D-GaIN/LPS
caspase-1
（43kd）
NLRP3
（110kd）
ASC
（24kd）
β-actin
（45kd）
The original western blotting bands of NLRP3, ASC, caspase1 and β-actin protein in liver tissue of ALF mcie (D-GaIN/LPS group and Rapamycin+D-GaIN/LPS group) and Controls.

## Slide 11
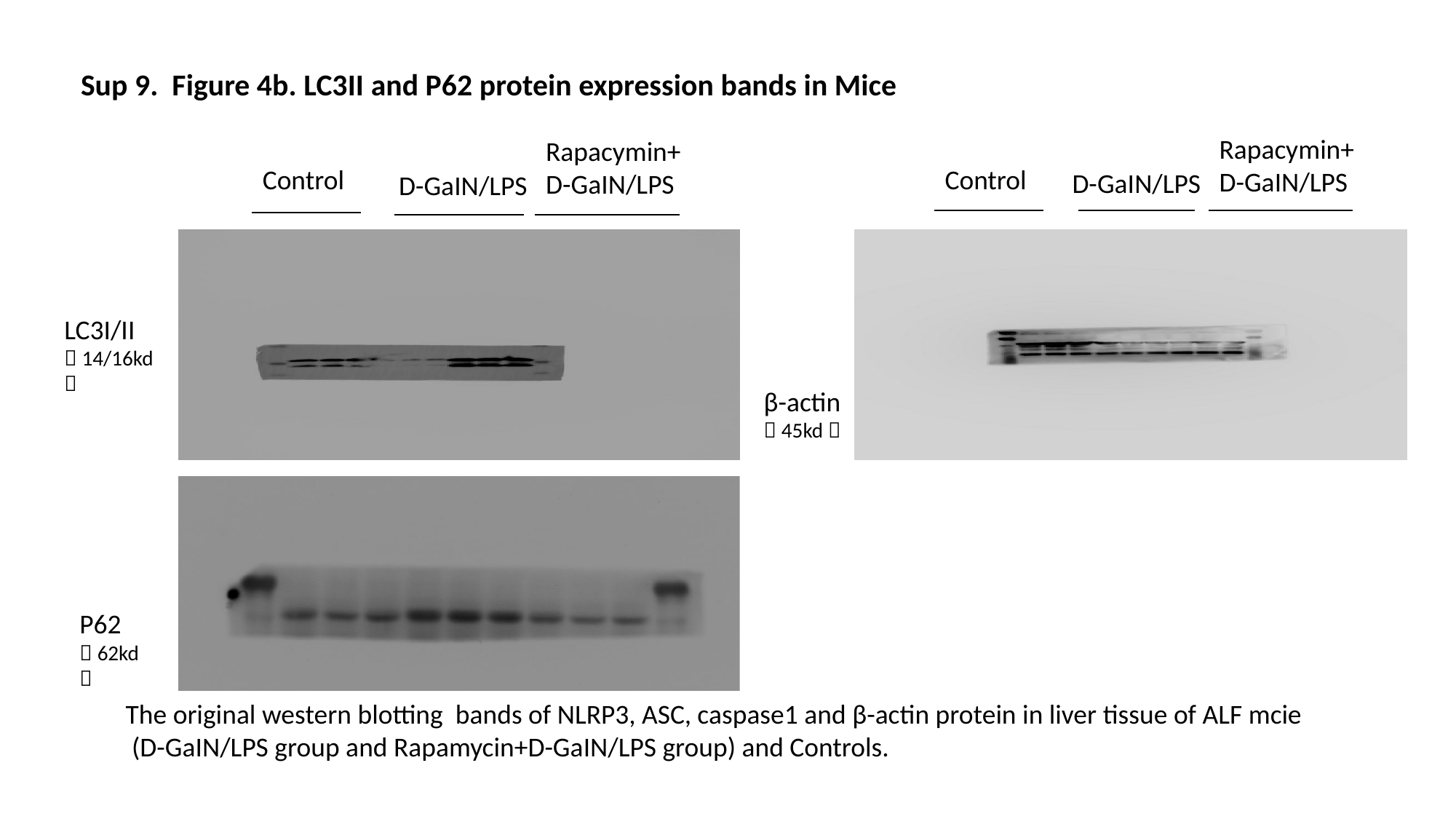

Sup 9. Figure 4b. LC3II and P62 protein expression bands in Mice
Rapacymin+
D-GaIN/LPS
Rapacymin+
D-GaIN/LPS
Control
Control
D-GaIN/LPS
D-GaIN/LPS
LC3I/II
（14/16kd）
β-actin
（45kd）
P62（62kd）
The original western blotting bands of NLRP3, ASC, caspase1 and β-actin protein in liver tissue of ALF mcie (D-GaIN/LPS group and Rapamycin+D-GaIN/LPS group) and Controls.

## Slide 12
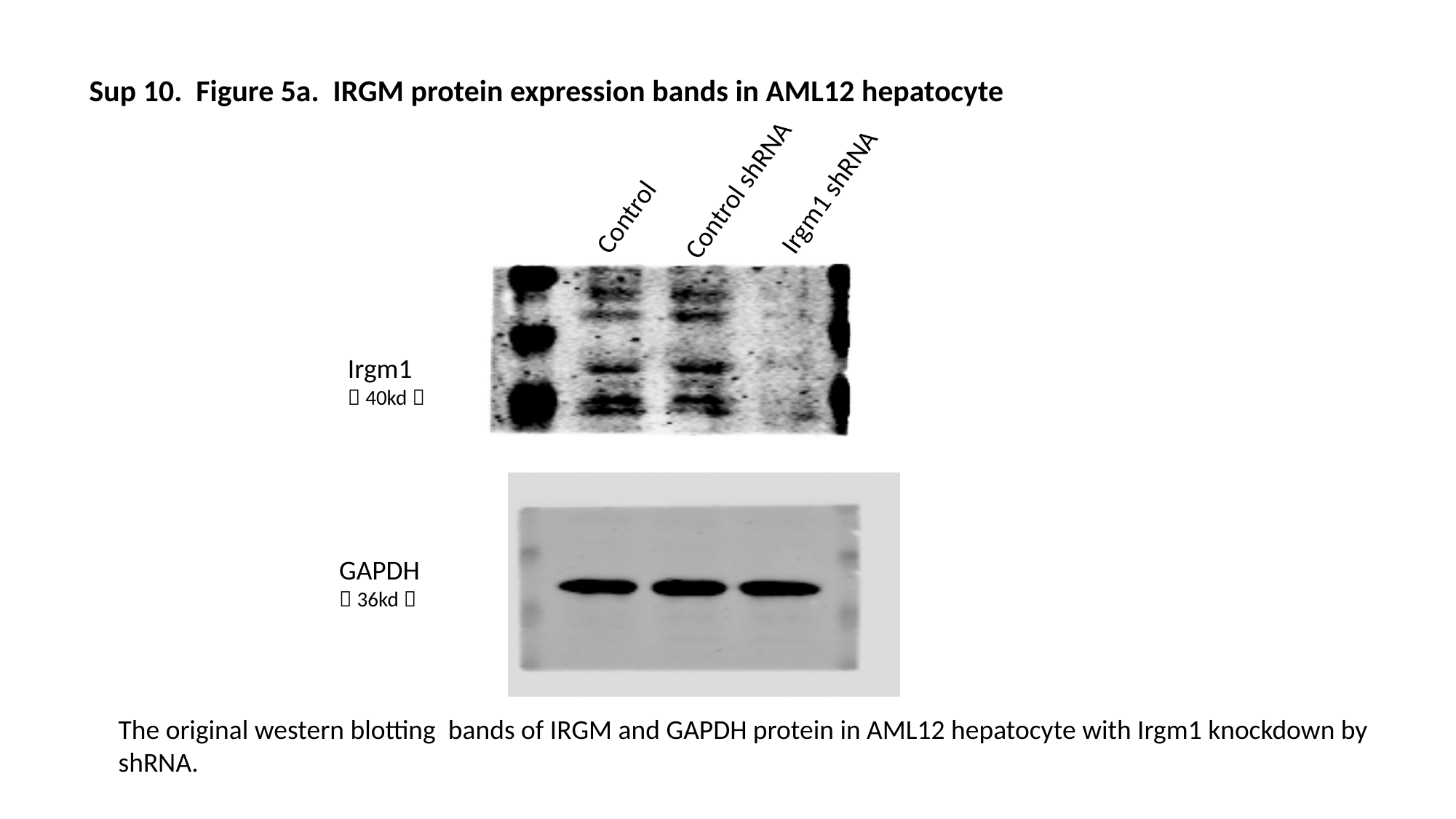

Sup 10. Figure 5a. IRGM protein expression bands in AML12 hepatocyte
Irgm1 shRNA
Control shRNA
Control
Irgm1
（40kd）
GAPDH
（36kd）
The original western blotting bands of IRGM and GAPDH protein in AML12 hepatocyte with Irgm1 knockdown by shRNA.

## Slide 13
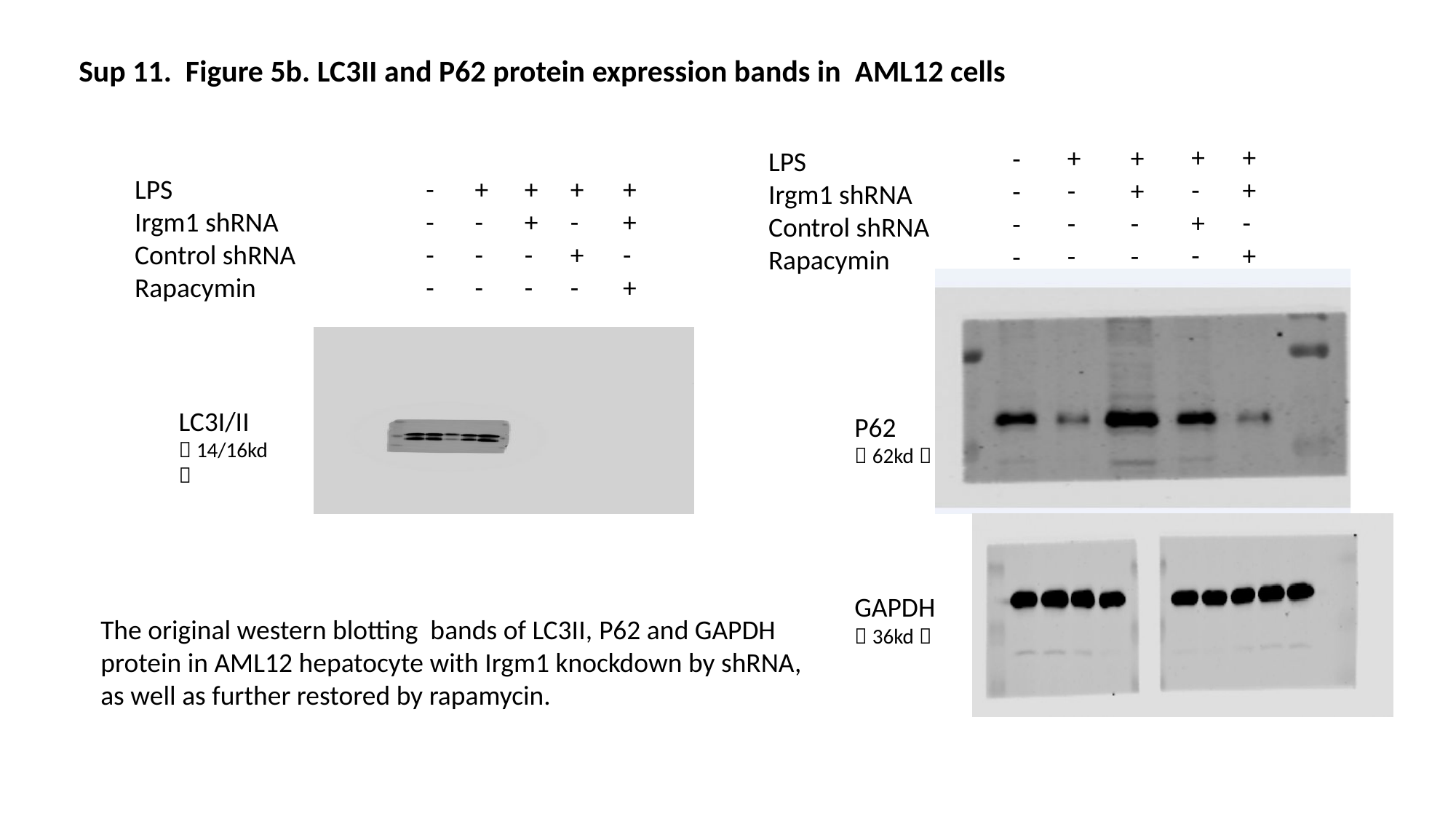

Sup 11. Figure 5b. LC3II and P62 protein expression bands in AML12 cells
+
-
+
-
+
+
-
+
+
-
-
-
+
+
-
-
-
-
-
-
LPS
Irgm1 shRNA
Control shRNA
Rapacymin
-
-
-
-
+
-
-
-
+
+
-
-
+
-
+
-
+
+
-
+
LPS
Irgm1 shRNA
Control shRNA
Rapacymin
LC3I/II
（14/16kd）
P62
（62kd）
GAPDH
（36kd）
The original western blotting bands of LC3II, P62 and GAPDH protein in AML12 hepatocyte with Irgm1 knockdown by shRNA, as well as further restored by rapamycin.

## Slide 14
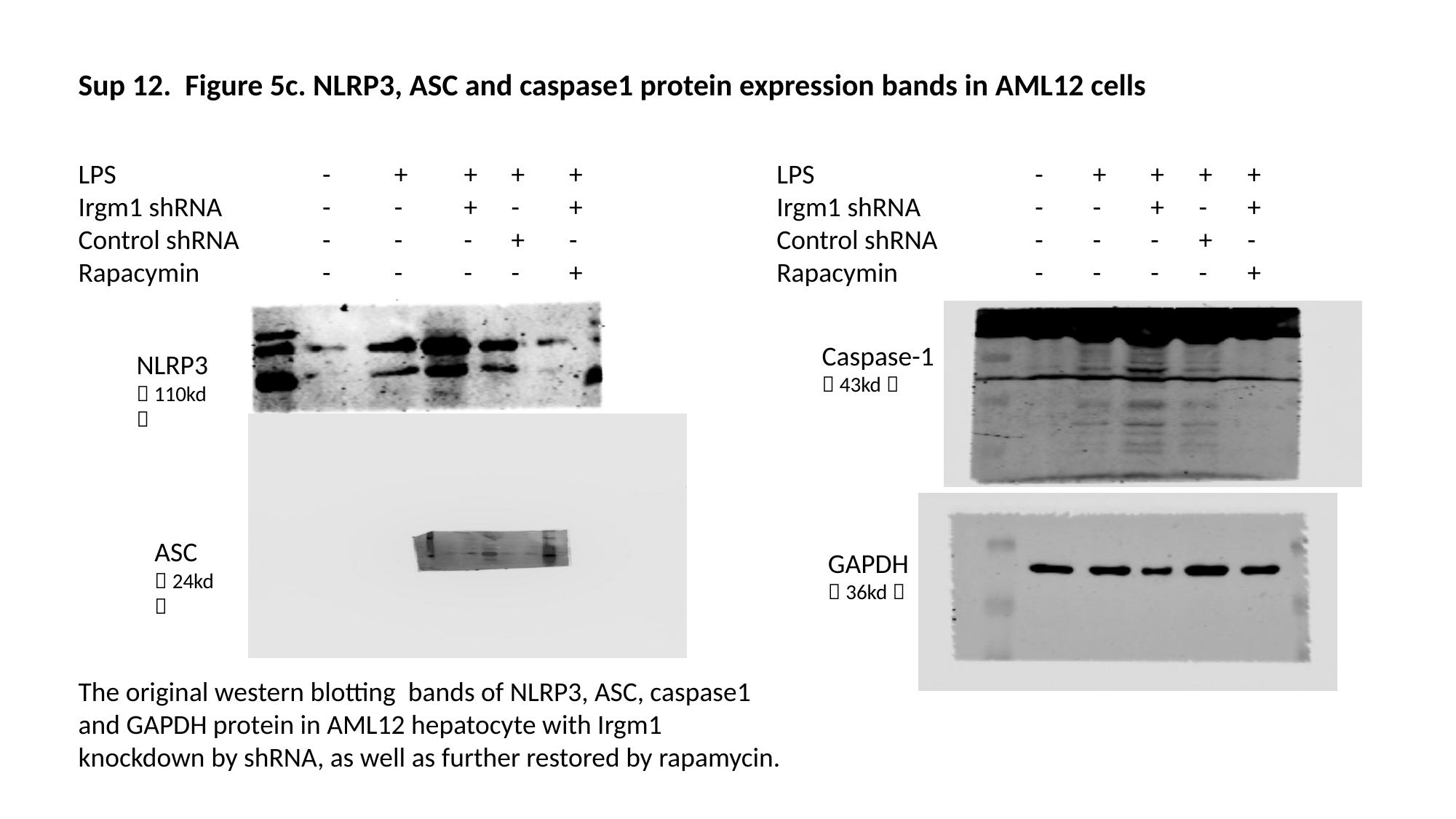

Sup 12. Figure 5c. NLRP3, ASC and caspase1 protein expression bands in AML12 cells
LPS
Irgm1 shRNA
Control shRNA
Rapacymin
-
-
-
-
+
-
-
-
+
+
-
-
+
-
+
-
+
+
-
+
LPS
Irgm1 shRNA
Control shRNA
Rapacymin
+
-
-
-
+
+
-
-
+
-
+
-
+
+
-
+
-
-
-
-
Caspase-1
（43kd）
NLRP3
（110kd）
ASC
（24kd）
GAPDH
（36kd）
The original western blotting bands of NLRP3, ASC, caspase1 and GAPDH protein in AML12 hepatocyte with Irgm1 knockdown by shRNA, as well as further restored by rapamycin.
